# Rationale and Design of Dual Antiplatelet Therapy in Patients with Coronary Multi‐Vessel Disease (DAPT‐MVD): A Multicenter, Randomized, Controlled Trial

**DOI:** 10.1002/clc.70049

**Published:** 2024-11-25

**Authors:** Jinwei Tian, Zhuozhong Wang, Yan Wang, Fan Wang, Yini Wang, Peng Zhao, Xinyu Hou, Xiang Peng, Maoyi Tian, Duolao Wang, Bo Yu

**Affiliations:** ^1^ Department of Cardiology The Second Affiliated Hospital of Harbin Medical University Harbin China; ^2^ State Key Laboratory of Frigid Zone Cardiovascular Diseases (SKLFZCD) Harbin Medical University Harbin China; ^3^ The Key Laboratory of Myocardial Ischemia Harbin Medical University, Ministry of Education Harbin China; ^4^ Heilongjiang Provincial Key Laboratory of Panvascular Disease Harbin China; ^5^ School of Public Health Harbin Medical University Harbin China; ^6^ Global Health Trials Unit Liverpool School of Tropical Medicine Liverpool UK

**Keywords:** clinical trial, coronary artery disease, drug‐eluting stent, dual antiplatelet therapy, multivessel disease

## Abstract

**Background:**

The optimal duration of dual antiplatelet therapy (DAPT) for patients with coronary multi‐vessel disease (MVD) who have received drug‐eluting stents (DES) remains unclear.

**Hypothesis and Methods:**

The Dual Antiplatelet Therapy in Patients with Coronary Multi‐Vessel Disease (DAPT‐MVD) study is a multicenter, open‐label, randomized controlled trial designed to assess the efficacy and safety of extended DAPT in MVD patients 12 months following DES implantation. We plan to enroll 8250 patients across approximately 100 sites in China. Participants will be randomized in a 1:1 ratio to receive either extended DAPT (75 mg clopidogrel plus 75–150 mg aspirin daily) or monotherapy (75–150 mg aspirin daily) beyond 12 months post‐DES implantation. The follow‐up period will last at least 12 months, with all potential endpoints adjudicated by a blinded Clinical Events Committee. The primary endpoint is major adverse cardiovascular and cerebrovascular events (MACCE), including cardiovascular death, nonfatal myocardial infarction, or nonfatal stroke.

**Results:**

As of April 2024, a total of 8250 participants have been enrolled in the study. The mean age of the enrolled patients was 60.5 ± 8.8years, with 5753 (69.7%) being men.

**Conclusions:**

The DAPT‐MVD study is the first large‐scale trial to evaluate the efficacy and safety of prolonged DAPT with clopidogrel plus aspirin beyond 12 months after DES implantation in MVD patients. The trial will provide novel insights into the optimal duration of DAPT for MVD patients (ClinicalTrials. gov ID: NCT04624854. Registered on 10/27/2020).

## Graphical abstract

DAPT‐MVD is the largest multicenter, parallel, open‐label, randomized controlled study focused on optimal antiplatelet therapy for MVD patients who have undergone DES implantation. The primary objective is to evaluate the efficacy and safety of prolonged DAPT with clopidogrel plus aspirin in these patients 12 months after DES implantation. Ultimately, the study aims to address the clinical conundrum regarding the optimal duration of DAPT in this challenging patient group with high ischemic risk.

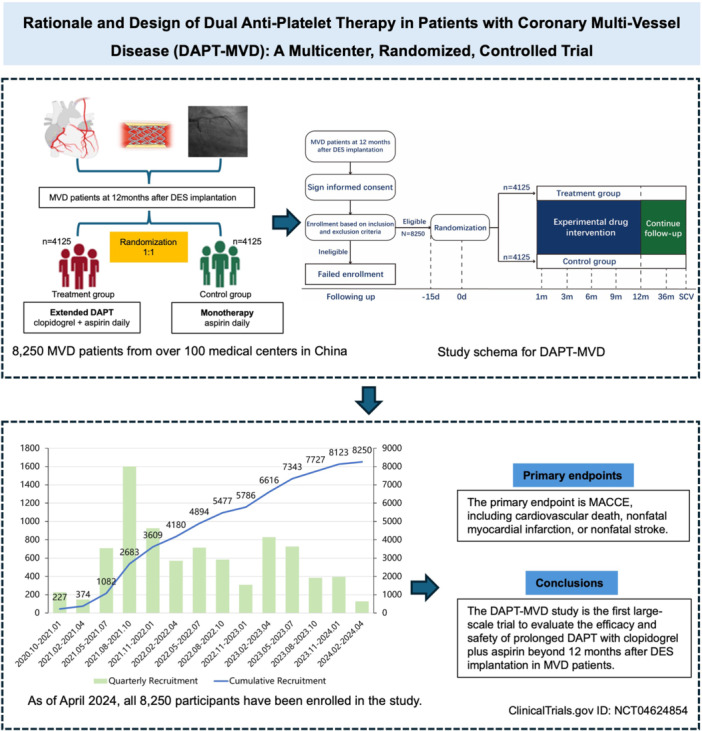

## 1 Introduction

The World Health Organization reported that 9 million people died of cardiovascular disease worldwide in 2019, accounting for 16% of the total deaths [1]. Aside from patients with single‐vessel disease, approximately 40%–65% of patients with coronary artery disease (CAD) are diagnosed with multivessel disease (MVD) [2, 3, 4, 5].

Compared to those with single‐vessel disease, patients with MVD have a more severe state of atherosclerosis in the vascular bed and tend to have worse clinical outcomes manifested as a higher cumulative incidence of recurrent atherothrombotic coronary events, revascularization, and adverse mortality [5, 6, 7]. With the increasingly widespread use of percutaneous coronary intervention (PCI) in the treatment of CVD, culprit vessels in MVD patients were effectively treated [8, 9].

Dual antiplatelet therapy (DAPT) with aspirin and an adenosine diphosphate (ADP) receptor blocker has been established as a standard therapy to prevent thrombotic arterial vessel occlusion after PCI [10]. Abundant evidence from clinical trials demonstrated a significant reduction in the risk of major adverse cardiovascular and cerebrovascular events (MACCEs) for 1 year with DAPT treatment relative to aspirin monotherapy following drug‐eluting stent (DES) implantation [11, 12, 13]. However, the duration of antithrombotic therapy for patients with MVD after PCI is still controversial.

The previous randomized controlled trial (RCT), PEGASUS‐TIMI 54, showed a dramatic reduction in the risk of MACCEs in patients with prior myocardial infarction (MI) and MVD treated with prolonged DAPT treatment (60 mg and 90 mg ticagrelor twice daily in patients treated with low dose aspirin) [14]. In consideration of the higher risk of bleeding due to the potent antiplatelet effect of ticagrelor, clopidogrel, another P2Y12 receptor inhibitor, can reduce net adverse clinical and cerebral events (NACCEs) and bleeding events compared with ticagrelor in a real‐world study [14, 15, 16]. Therefore, clopidogrel‐based DAPT is theoretically more suitable for long‐term antiplatelet therapy among patients with MVD following DES implantation.

Given these, we designed the Dual Antiplatelet Therapy in Patients with Coronary Multi‐Vessel Disease (DAPT‐MVD) trial to evaluate whether extending DAPT with clopidogrel and aspirin for 12 months would improve the long‐term prognosis for MVD patients 12 months after DES implantation. The DAPT‐MVD trial aims to provide novel insights and robust clinical evidence for managing targeted patients. Given the possibility of increased bleeding risk, it is vital to prudently evaluate the preferable effect of prolonged DAPT [17, 18]. Hence, for MVD patients at 12 months after DES implantation, it is essential to weigh the beneficial effects of prolonged DAPT treatment against an increased bleeding risk.

## 2 Methods

### 2.1 Study Design and Population

**Figure 1:**
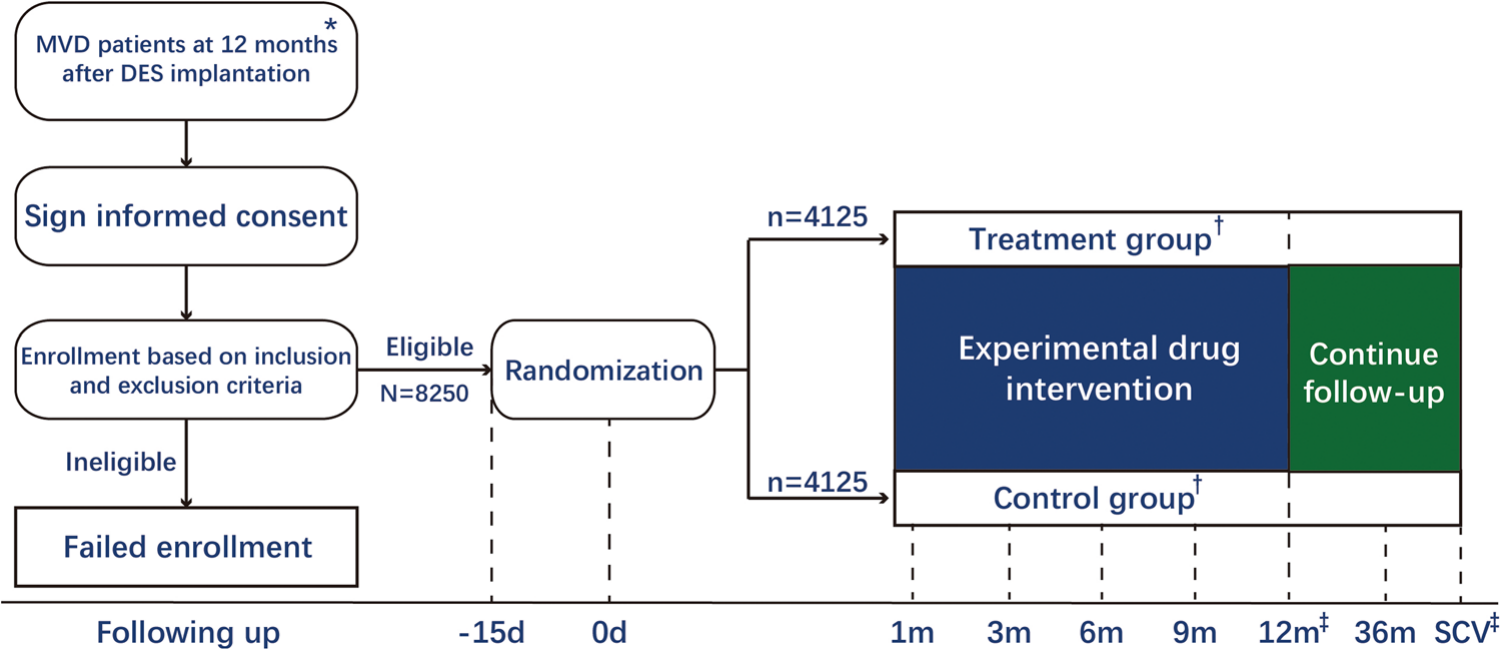
Study schema for DAPT‐MVD. *The time window is 12 ± 3 months; ^†^The treatment group received clopidogrel plus aspirin dual antiplatelet therapy within 12 months, while the control group received aspirin monotherapy within 12 months. Other treatments were given according to the clinical routine protocol; ^‡^The final follow‐up endpoint of the overall study is 12 months after the randomization of the last subject. d, Day; DES: Drug‐eluting stent; m, Month; MVD, Multivessel coronary artery disease; SCV, Site close‐out visit.

The DAPT‐MVD study (ClinicalTrials. gov unique identifier NCT04624854) is a multicenter, open‐label, randomized, controlled trial aimed to evaluate the efficacy and safety of prolonged DAPT treatment in MVD patients beyond 12 months after DES implantation. The recruitment period for the study is planned to range from November 01, 2020, to the estimated primary completion date of April 30, 2024. A flow diagram of the patient recruitment process is shown in Figure 1. The primary hypothesis is that prolonged DAPT therapy will reduce the incidence of MACCE (including cardiovascular death, nonfatal myocardial infarction, or nonfatal stroke).

### 2.2 The Participants‐Inclusion and Exclusion Criteria

After a median of 12‐month DAPT run‐in phase after DES implantation, a total of 8250 eligible patients recruited from ≥ 100 cardiology centers across China and randomized to continue a prolonged 12‐month DAPT treatment (75 mg clopidogrel plus 75–150 mg aspirin daily) or a monomer treatment (75–150 mg aspirin daily) until study completion. The enrolled subjects will have angiographically confirmed MVD with stenosis of ≥50% in more than two major epicardial coronary arteries and stenosis in the left main coronary artery ≤30% by visual assessment. Additional details of the inclusion and exclusion criteria are shown in Table 1.

**Table 1:** Inclusion/exclusion criteria.

Inclusion	Exclusion
Aged 18–75 years old (inclusive). Patients with MVD who underwent DES implantation for 12 months. Patients have been treated with aspirin and can tolerate aspirin at doses of 75–150 mg/day as maintenance therapy during the study period. Patients have signed informed consent.	Planned to use of ADP receptor blockers (eg, clopidogrel, ticagrelor, and ticlopidine), dipyridamole, or cilostazol. Contraindication to ADP receptor blockers or aspirin. Anticoagulants were planned to be used during the study period. Planned major cardiac or noncardiac surgery during the study period. Concomitant oral or intravenous therapy with CYP2C19 medium or strong inhibitors. Known severe liver disease (ALT/AST is three times above normal). Subjects with renal failure who required or anticipated dialysis during the study period. Platelet count < 50 × 10^9^/L. Patients with a. A history of intracranial bleeding or ischemic stroke at any time; b. A central nervous system tumor or intracranial vascular abnormality (e.g., aneurysm, arteriovenous malformation) at any time; c. Intracranial or spinal cord surgery within five years. Pregnancy or lactation or planned to be pregnant during the study period. Life expectancy < 1 year. In the investigator's opinion, any condition would make it unsafe or unsuitable for the patient to participate in this study (e.g., active malignancy other than squamous or basal cell skin cancer). Concern for the inability of the patient to comply with study procedures and/or follow‐up (e.g., alcohol or drug abuse). Participation in another clinical study and did not reach the major endpoint. Involvement in the planning and/or conduct of the study.

### 2.3 Randomization and Treatment

After assessment for study eligibility requirements, patients will be assigned on a 1:1 basis to either the treatment group or the control group using a completely random method. Randomization will be conducted by a central computer network system. All eligible patients enrolled will receive 75–150 mg aspirin daily, and the treatment group will be recommended to take 75 mg clopidogrel daily for an additional 12 months from randomization. The subjects will receive experimental drug (the dose for 6 months, including the window period) at the research centers and their 6‐month follow‐up. As the study population is stable, no loading dose will be administered. Researchers may adjust the dose and regimen for antiplatelet medicines when adverse events occur in subjects. Other concomitant medications must be taken under the guidance. Any adjustment and distribution of study drugs should be recorded on the CRF form.

### 2.4 Endpoints

A Clinical Events Committee (CEC) will evaluate all potential endpoints, with all committee members unaware of the trial group assignments. The primary endpoint is to determine whether extending DAPT with clopidogrel and aspirin for 12 months reduces the incidence of the composite efficacy endpoints of cardiovascular death, nonfatal MI, or nonfatal stroke (MACCEs) compared with aspirin monotherapy.

The secondary endpoints are to evaluate the effect of extending DAPT with clopidogrel and aspirin for 12 months on the incidence of all‐cause mortality, cardiovascular death, nonfatal MI, nonfatal stroke, net clinical adverse events (NACEs, including MACCEs and type 2–5 hemorrhage defined by Bleeding Academic Research Consortium [BARC]), cardiovascular death or hospitalization caused by cardiovascular or cerebrovascular thrombotic events (MI, stroke, emergency revascularization, unstable angina or transient ischemic attack (TIA), emergency revascularization, any repeat revascularization and definite/probable stent thrombosis (ST) individually in such patients.

The safety endpoints are to assess whether extending DAPT with clopidogrel and aspirin for 12 months increases the rate of clinically relevant bleeding events (BARC type 2–5) and the rate of major bleeding events (BARC type 3–5) relative to aspirin monotherapy.

### 2.5 Follow‐Up

After randomization, participants will receive the assigned experimental drug treatment according to their group and have their treatment adherence and concomitant medication assessed at 1, 3, 6, 9, and 12 months of follow‐up. The experimental drug will be discontinued 12 months post‐enrollment. Participants may stop treatment at any time, and the experimental drug will also be discontinued if the researchers believe continued treatment poses potential risks. Then, after discontinuing the experimental drug 12 months post‐enrollment, subjects will continue to complete a 36‐month visit and the study close‐out visit (SVC) (12 months after the enrollment of the last patient). We will follow up on all participants’ potential endpoints and adverse events until death or the end of the follow‐up period.

### 2.6 Sample Size Calculation

Based on data extracted from the PEGASUS‐TIMI 54 study, the expected annual rate of MACCEs is estimated to be 5% in the control arm [19]. It is anticipated that prolonged DAPT treatment in patients with MVD will reduce the target relative risk by 20% (i.e., the hazard ratio [treatment/control] is equal to 0.8) and the per‐year event rate by approximately 4% for the primary endpoint in those populations [20, 21]. Moreover, a two‐sided test is set at the 5% significance level, and 80% power is designed to reject the null hypothesis. The enrollment duration is estimated to be 24 months, and the follow‐up time is at least 12 months (with an expected median follow‐up time of 24 months). The enrollment progress in both groups was consistent, and eligible patients will be randomized on a 1:1 basis according to the protocol. Considering a 5% dropout rate and an expected noncompliance (switching groups) rate in those groups will be 3%. Therefore, the DAPT‐MVD trial is required to enroll at least 8250 subjects (4125 in each arm).

### 2.7 Statistical Considerations

All potential endpoints will be adjudicated by the CEC and included in the efficacy analysis in the intention‐to‐treat (ITT) set, and a cross table of judgment results for the events will be listed. Subjects may develop more than one event during the study. For the composite endpoint, only the first event that occurs will be included in the time‐to‐event endpoint analysis. Unless otherwise specified, all statistical tests will be two‐sided and conducted at a 5% significance level. The detailed descriptions are summarized in Supporting Information S1: Appendix 1.

The primary endpoint will be the time from randomization to the first occurrence of its component. Initially, the Kaplan–Meier method will be employed to estimate the cumulative incidence of the primary endpoint and a log‐rank test will be conducted to compare survival curves between the treatment arms. Furthermore, a Cox proportional hazards model will be utilized to compare the hazard of having a primary outcome between the prolonged DAPT arm and control arm, providing a hazard ratio along with a 2‐sided 95% confidence interval (CI) to measure the treatment difference. In addition, the win‐ratio method for the hierarchical composite outcome will be performed as a supplementary analysis (Supporting Information S1: Appendix 1) [22]. All secondary time‐to‐event endpoints will be analyzed similarly as for the primary endpoint.

All safety analyzes will be based on the safety set. Descriptive statistical methods will be used to summarize the study drug exposure, the causes and times of study drug discontinuation, concomitant drugs, protocol deviations, clinical laboratory data, vital signs, and physical examination results in each treatment group. Patients not having suffered any bleeding event in the given category will be censored at the earliest of 7 days after the last dose of the study drug, death, last contact, or withdrawal of consent.

Covariate adjustment analysis aims to control for possible imbalances in the covariates, as listed in Table 2. The Cox model will be used for the covariate‐adjusted analysis, and the adjusted hazard ratio with its 95%CI will be estimated. In addition, subgroup analysis will be performed to assess the homogeneity of treatment effects across the categories of pre‐specified subgroup variables. Detailed statistical analyzes will be described in the statistical analysis plan. The trial results will be reported following the CONSORT guidelines for reporting randomized trials.

**Table 2:** List of covariates used in the covariate adjustment analysis.

Characteristics	Categories
Age	< 60, ≥ 60 year
Gender	Male, Female
Body mass index	< 28, ≥ 28 kg/m^2^
Diabetes	Yes, No
Hypertension	Yes, No
Hyperlipidemia	Yes, No
History of smoke	Yes, No
History of alcohol	Yes, No

## 3 Results

**Figure 2:**
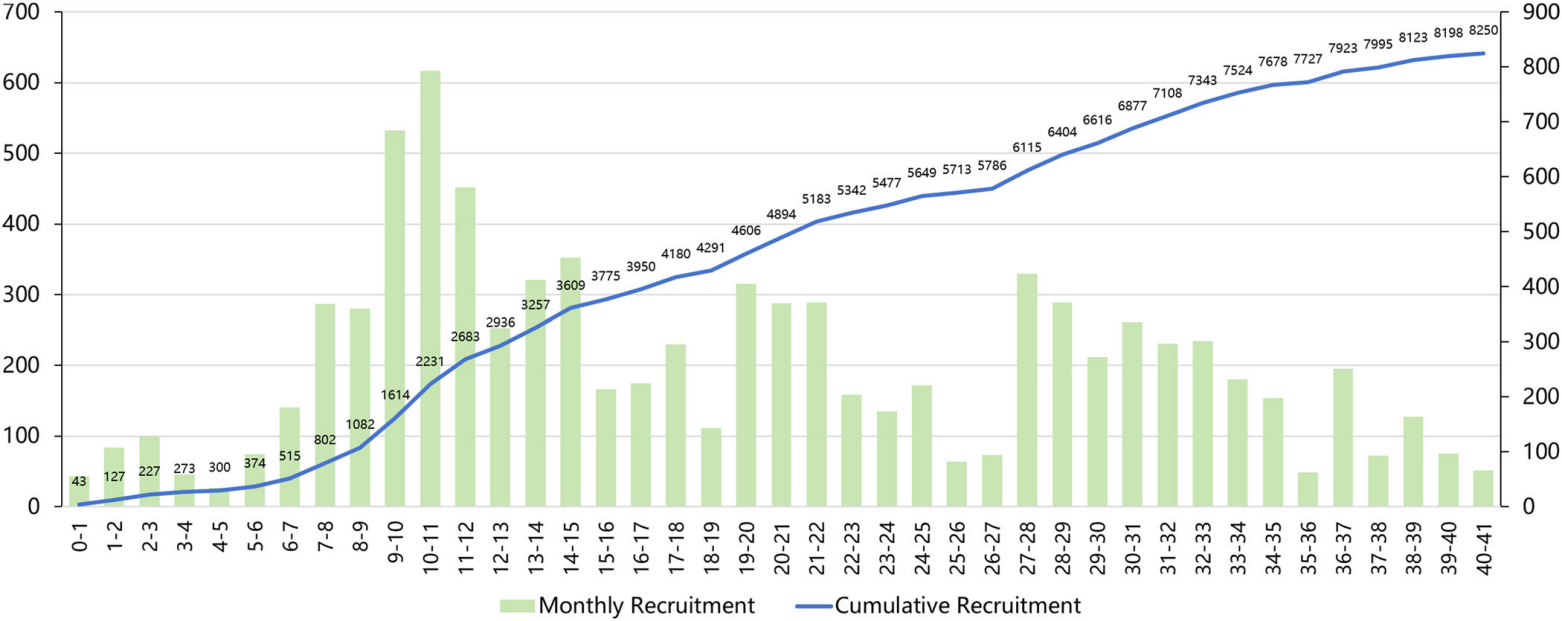
Monthly and cumulative recruitment.

The first patient was enrolled on October 28, 2020. By April 2024, 9127 participants had been screened for the study, with 877 deemed ineligible, leading to the enrollment of 8250 patients (Figure 2). The mean age of the enrolled patients was 60.5 ± 8.8 years, with 5753 (69.7%) being men (Table 3).

**Table 3:** Preliminary data for patients enrolled as of April 2024.

Characteristics	*N* = 8250
Age, years	60.5 ± 8.8
Male gender, *n* (%)	5753 (69.7%)
Height, cm	168.0 ± 7.3
Weight, kg	71.6 ± 10.3
History of myocardial infarction, *n* (%)	2806 (34.0%)
History of heart failure, *n* (%)	732 (8.9%)
Systolic blood pressure, mmHg	131.4 ± 13.5
Diastolic blood pressure, mmHg	80.2 ± 9.4
History of hypertension, *n* (%)	4414 (53.5%)
History of diabetes, *n* (%)	2323 (28.2%)
Time since most recent PCI, day	378.6 ± 19.8

## 4 Discussion

The main findings of this study can be summarized as follows.

1 DAPT‐MVD is the largest multicenter, parallel, open‐label, randomized, and controlled study focusing on optimal antiplatelet therapy for MVD patients who underwent DES implantation combined with the latest cardiovascular risk management guidelines.
2 This study is planned to recruit 8250 MVD patients after DES implantation 12 months prior at one of 100 different centers with the intention to continue prolonged the duration of 75 mg clopidogrel and 75 to 150 mg aspirin‐based DAPT by 12 months. During the follow‐up period, the first occurrence of any MACCE event (a composite event of cardiovascular death, nonfatal MI, or nonfatal stroke) will be collected to evaluate the effect of prolonged DAPT compared with aspirin treatment alone.
3 The safety endpoints are defined as clinically relevant bleeding events (BARC, defined as type 2–5) and major bleeding events (BARC, defined as type 3–5) to observe the safety of prolonged DAPT in patients with MVD.

In the era of PCI with significant progress, the gap in clinical outcomes between PCI and coronary artery bypass grafting (CABG) has narrowed over time. Accordingly, it is reported that the number of PCIs performed for MVD increased by 56% per year between 2001 and 2006; moreover, the number of CABG surgeries declined by 24% and subsequently fell at an annual rate of 5% [23]. After MI and/or PCI, DAPT with a P2Y12 inhibitor and aspirin is the mainstay to prevent thrombosis. However, owing to the presence of high plaque load and extensive ischemia risk, a heightened risk of adverse events remains in MVD patients after administration of contemporary treatment therapies, with a 3.1% cumulative risk of all‐cause death, 1.9% cumulative risk of MI and 0.7% cumulative risk of stroke [20]. Moreover, whether prolonged DAPT based on 12 months of treatment after PCI is beneficial remains debatable. Despite the high prevalence and mortality of MVD, a paucity of evidence‐based medicine evidence exists regarding optimal DAPT management among particular patients. The MASTER DAPT study, a multicenter randomized controlled trial led by Marco Valgimigli et al. [24], was designed to compare the NACEs in patients at higher risk of bleeding after implantation of biodegradable polymer‐coated sirolimus‐eluting stents with a shorter duration of dual antiplatelet therapy. The study showed that 1 month of dual antiplatelet therapy was non‐inferior to the continuation of treatment for at least two additional months with regard to the occurrence of net adverse clinical events and major adverse cardiac or cerebral events; abbreviated therapy also resulted in a lower incidence of major or clinically relevant nonmajor bleeding. This suggests that future research should focus on individualized antiplatelet therapy. Few prospective studies are concentrated on antiplatelet therapy after revascularization in MVD patients. Thus, it becomes increasingly urgent to answer the ultimate question, “What is the ideal duration of DAPT for patients with MVD following DES implantation?” and further, “How can the duration of DAPT be adjusted after PCI with MVD based on the patient's bleeding‐ischemia risk to reduce future ischemic events with less nonfatal bleeding while obtaining greater clinical benefits?”

According to the *2020 ESC Guidelines for the Management of Acute Coronary Syndromes in Patients Presenting without Persistent ST‐segment Elevation*, it is recommended for patients with non‐ST‐segment elevation acute coronary syndromes (NSTE‐ACS) to receive DAPT consisting of aspirin and a P2Y12 receptor inhibitor 12 months following PCI unless there are contraindications (I, A) [25]. Given that patients with MVD are at a high ischemic risk, the occurrence of ischemic events has a greater impact on their prognosis than bleeding events. Therefore, such patients may appear to derive a net clinical benefit from prolonged DAPT treatment.

The CHARISMA trial tested the effect of clopidogrel versus placebo on an aspirin background among patients with prior myocardial infarction, ischemic stroke, or symptomatic peripheral arterial disease. During the follow‐up for a median of 27.6 months, patients with clopidogrel plus aspirin benefited significantly from intensifying antiplatelet therapy compared with the controls. Clopidogrel significantly reduced the risk of MACCE (7.3% vs 8.8%, *p* = 0.01) and did not significantly increase the risk of severe bleeding (1.7% vs. 1.5%, *p* = 0.50) [26]. In addition, a large‐scale meta‐analysis of 6 randomized controlled trials, including 1680 patients who underwent complex PCI, showed that long‐term DAPT (≥ 1 year) versus short‐term DAPT (3–6 months) yielded significant reductions in MACEs (adjusted hazard ratio is 0.56) and coronary thrombotic events(adjusted hazard ratio is 0.57) [27].

However, the extension of DAPT increased the risk of bleeding, making the clinical decision‐making process on the optimal duration of DAPT more challenging. The DAPT study showed that continuation of thienopyridine‐plus‐aspirin therapy beyond 18 months reduced the risks of MACCE (4.3% vs. 5.9%; *p* < 0.001) and ST (0.4% vs. 1.4%; *p* < 0.001) compared with aspirin therapy alone among patients who had no severe ischemia or bleeding events during the first year of follow‐up after DES implantation. The clinical benefit of prolonged DAPT was tempered by an increase in moderate or severe bleeding events (2.5% vs. 1.6%, *p* = 0.001) [28].

At present, it may be reasonable that patients with ACS take prolonged DAPT beyond 12 months after coronary stent implantation if patients can tolerate DAPT without bleeding (IIb, class A) [29]. However, evidence and clear consensus to support the benefit of prolonged DAPT beyond 12 months following DES implantation in MVD patients still need to be improved. There is no adequately powered, randomized trial to determine how to prolong the DAPT course further to achieve a favorable balance between the increased risk of bleeding and the prevention of ischemic events in such patients. In addition, the current large clinical trials have mainly enrolled patients who underwent DES implantation with extensive clinical manifestations, and those trials were not explicitly focused on patients with MVD. The primary objective is to evaluate the effect of prolonged DAPT with clopidogrel plus aspirin in MVD patients 12 months after DES implantation. More importantly, it also investigates whether prolonged DAPT can increase ischemic benefits within an acceptable range for bleeding burden. This DAPT‐MVD study will provide substantial evidence for evidence‐based medicine and treatment strategies in patients with MVD.

## 5 Limitations

This study has several limitations. First, the open‐label design introduces potential observational bias; however, this was mitigated by keeping the principal investigators blinded to group assignments, with data analysis conducted by an independent statistician. Second, the widespread transmission of COVID‐19 in China disrupted the trial recruitment process. To ensure adequate participant enrollment, the recruitment period was extended by an additional 18 months, and the number of study centers was increased. Third, there is a regional disparity in the trial population, with more participants from northern China than the south. However, this imbalance aligns with the higher prevalence of coronary artery disease in northern regions, which may enhance the generalizability of the findings.

## 6 Conclusion

The DAPT‐MVD study is the first large‐scale trial to evaluate the effectiveness and safety of prolonged DAPT with clopidogrel and aspirin for another 12 months beyond the completion of 12 months of DAPT in MVD subjects following DES implantation. Upon completion, the results are expected to significantly influence therapeutic decision‐making about the optimal pharmacotherapy strategy in MVD by offering an equilibrium point between protection against ischemia events and the risk of bleeding. The study will ultimately illustrate the clinical conundrum regarding the optimal duration of DAPT among such challenging patient groups with high ischemic risk.

## Conflicts of Interest

The authors declare no conflicts of interest.

## Supporting information

Supporting information.

## Data Availability

All the investigators involved in the trial will have access to the full data set. We support the reuse of scholarly data and intend that the data to be collected in this trial may contribute beyond our actions to the knowledge of pharmacotherapy management of MVD. First, we will provide in writing the final results of the research for each participant. Second, we have obtained ethical consent from participants as well as research ethics board approval to share deidentified data after trial completion through presentation in congresses and publications in journals. The DAPT‐MVD study set up a Data and Safety Monitoring Board(DSMB). If the DSMB expresses concern about the safety of the study treatment plan, the study may be terminated prematurely.
