# Supplementary material for: Rationale and Design of Dual Antiplatelet Therapy in Patients with Coronary Multi‐Vessel Disease (DAPT‐MVD): A Multicenter, Randomized, Controlled Trial
Source: Clin Cardiol. 2024 Nov 25;47(12):e70049. doi: 10.1002/clc.70049 (PMC11589165; doi:10.1002/clc.70049)
Supplement: Supplementary file 1 — Supporting information. [file CLC-47-e70049-s001.docx]

# 1. Analysis populations

# 1.1 Intention-to-Treat (ITT) population.

The Intention-to-Treat (ITT) set includes all subjects who will be randomized, regardless of whether they have discontinued the study drug. The ITT set is the main population in which the endpoints of this trial will be evaluated. Patients will be analyzed according to their randomization scheme, regardless of whether their outcome event occurs before or after treatment.

# 1.2 Per protocol (PP) population.

The PP set is a subset of the ITT set and includes subjects with no major protocol deviations in the ITT population. These protocol deviations may seriously affect the primary efficacy endpoint of this study; therefore, subjects with major protocol deviations must not be included in this analysis population. The definition of a major protocol deviation will be determined after discussion at the blinding data audit meeting. At that meeting, the degree of compliance in the blinded state and possible protocol violations will be used to confirm the situation regarding major protocol violations. The PP set will be used to analyze endpoints and is the secondary data set for endpoint evaluation in this trial. The study population excluded from the PP set will be confirmed before database locking.

# 1.3 Safety population.

The Safety set (SS) includes all patients undergoing at least one safety evaluation after randomly assigned treatment. Subjects who need to be rejected from the ITT set and the PP set will be identified at a data review meeting.

# 1.4 Pre-scheduled stopping of study participants and use of data.

In case the intervention was stopped before the pre-scheduled end, either by the decision of the study patients themselves or by the study team, and data was collected after stopping the intervention, the information will be included in the ITT set. Subjects who need to be rejected from the ITT set and the PP set will be identified at a data review meeting.

# 2. General considerations for data analyses

Clinical events reported after database lock will not be included in the primary efficacy and safety analysis. These events will be recorded in the CRF, and may be included in the sensitivity analysis. Demographic characteristics, relevant risk factors, and specific histories of other diseases or surgery will be included in the ITT set for purposes of descriptive statistics and analysis.

# 2.1 Reporting guidelines

We will follow the Consolidated Standards of Reporting Trials (CONSORT) 2010 statement: updated guidelines for reporting parallel group randomized trials (http://www.consort-statement.org/).

# 2.2 Binary outcomes

For binary endpoints, the following will be calculated: 1) Crude (unadjusted) prevalence data (numerator, denominator, % per arm); 2) Crude risk ratio (RR) (95% CI); 3) *P*-value for the crude RR.

# 2.3 Continuous outcomes

Differences in continuous endpoints will be assessed by linear regression analysis. For continuous endpoints, the following will be calculated: 1) Crude mean and SD per arm; 2.) Crude mean difference (95% CI); 3) *P*-value for crude mean difference.

# 2.4 Time-to-event outcomes

Definitions of primary endpoint events and secondary endpoint events. The analysis methods to be mainly used include:

1. The Kaplan-Meier method will be used to display the cumulative event rates by treatment arm.
2. The log-rank test will be used to compare the curves of cumulative event rates between the treatment group and control group.

3) The Cox proportional hazard regression model will be used to estimate the treatment effect. A hazard ratio (HR) and its two-sided 95% confidence interval will be calculated.

Primary analyses will be on the intention to treat population and supportive analyses will be performed on the per protocol population.

# 2.5 Data summaries

The mean, standard deviation, minimum, and maximum values will be used to describe results for continuous variables with a normal distribution. The median, minimum, and maximum values, and the 25^th^ and 75^th^ percentiles, will be used to describe results for continuous variables with an abnormal distribution. The treatment-specific number and percentage of subjects in each category will be presented to describe results for categorical parameters.

# 2.6 Group comparison analysis

The independent two samples t-test will be used for inter-group comparisons of continuous variables with a normal distribution, and the Wilcoxon rank sum test will be used for inter-group comparisons of continuous variables with an abnormal distribution. The chi-square (χ^2^) test will be used for inter-group comparisons of categorical variables, and when the theoretical frequency is < 1 for cells > 25%, Fisher exact test will be used. Analysis of variance in time series will be performed for repeated measurements data, and logistic regression model will be constructed for the multivariable analysis of binary outcome.

Unless otherwise specified, statistical analysis results and confidence intervals will be expressed in the form of a two-sided test. *P*-values will be displayed to 3 decimal places, and a *P*-value < 0.05 will indicate statistical significance. Multiple corrections will not be performed for the confidence intervals of primary and secondary endpoint events, because they will be used as a measure for descriptive interpretation and accuracy. The *P*-values and widths of the confidence intervals will not be adjusted for multiplicity, so should not be used to infer definitive treatment effects for secondary outcomes. Primary and secondary efficacy variables will be included in the validation trial procedure. SAS (9.2 or a higher version) and/or R (3.6.2 or a higher version) will be used for the analyses in this study.

# 2.7 Missing data

Missing data will be dealt with differently for the endpoints and the independent covariate variables as follows.

# 2.7.1 Endpoints

Missing data will not be imputed during the primary and secondary endpoints analyses.

# 2.7.2 Covariates

Missing baseline covariates will be imputed using simple imputation methods in the covariate adjusted analysis based on the covariate distributions, should the missing values for a particular covariate be less than 5%. For a continuous variable, missing values will be imputed from random values from a normal distribution with mean and SD calculated from the available sample. For a categorical variable, missing values will be imputed from random values from a uniform distribution with probabilities *P*_1_, *P*_2_, …, and *P*_k_ from the sample. If the missing values for a covariate are ≥5% then they will be imputed using Markov chain Monte Carlo (MCMC) methods using SAS PROC MI. Seed for the imputation is set as 128.

**2.8 Subgroup analysis.** Subgroup analyses will be performed for the primary endpoint in this study and will include pre-specified variables that are likely to be prognostic factors for the primary outcome, as predefined based on the literature, as well as variables that are possibly prognostic factors for the primary outcome (details are shown in Table 2). We will include an interaction between the treatment group and each of the covariates in Table 2 in a separate Cox model to assess whether the effect of the intervention on the primary endpoints is homogenous across the categories of each subgroup variable.

**2.9** **Sensitivity analysis.** A logarithmic cumulative hazard map will be used to visually evaluate the proportional hazard assumption for the treatment group variable. The effects of any deviation from the proportional hazard will be discussed when presenting the analysis results. A sensitivity analysis will be performed to evaluate the possible effects of data and information censoring, and the strategy for that analysis is as follows. Sensitivity analysis of the primary composite endpoint will also include analysis with

1) Cardiovascular death replaced with all-cause mortality, including vital status information from patients who have withdrawn consent.

2) To assess the effect of the center on the treatment effect, a Cox regression model with frailty will be employed.

**2.10 Win ratio analysis.** The primary endpoint (MACCE) has been analyzed as the time from the date of randomization to the first occurrence of any components (cardiovascular death, nonfatal myocardial infarction, or nonfatal stroke). This method has an inherent limitation in that it emphasizes each patient’s first event, which is often the outcome of lesser clinical importance. To overcome this problem, we will use the win ratio for reporting composite endpoints. Patients in the new treatment and control groups are formed into all possible pairs. Consider the primary composite endpoint, e.g., the composite event of cardiovascular death, nonfatal myocardial infarction, or non-fatal stroke. The new treatment patient is labeled a ‘winner’ or a ‘loser’ for each pair, depending on who has a CV death first. If that is not known, only then are they labeled a ‘winner’ or ‘loser’ depending on who had a nonfatal myocardial infarction first. If that is not known, only then are they labeled a ‘winner’ or ‘loser’ depending on who had a non-fatal stroke first. Otherwise, they are considered tied. The win ratio is the total number of winners divided by the total number of losers. A 95% confidence interval and *P*-value for the win ratio will be calculated.

**2.11 Safety analysis.** The number (%) of patients with an event and number of an adverse event, related adverse events (AEs), and serious adverse events (SAEs) will be calculated in both groups, and the χ2 test or Fisher's exact test will be used to compare the proportion in the two groups. All completed laboratory examinations will be listed in the form of a cross table before and after treatment (based on the investigator's judgment of their clinical significance), and subjects who have normal results before treatment and abnormalities after treatment or have abnormalities before treatment and aggravated abnormalities after treatment, will be listed and the changes will be described.
